# Evidence for morphological composition in compound words using MEG

**DOI:** 10.3389/fnhum.2015.00215

**Published:** 2015-04-28

**Authors:** Teon L. Brooks, Daniela Cid de Garcia

**Affiliations:** ^1^Department of Psychology, New York UniversityNew York, NY, USA; ^2^Department of Anglo-Germanic Languages, Universidade Federal do Rio de JaneiroRio de Janeiro, Brazil

**Keywords:** compounds, MEG, left anterior temporal lobe (LATL), word naming, morphology, semantic transparency, morphological decomposition, morphological composition

## Abstract

Psycholinguistic and electrophysiological studies of lexical processing show convergent evidence for morpheme-based lexical access for morphologically complex words that involves early decomposition into their constituent morphemes followed by some combinatorial operation. Considering that both semantically transparent (e.g., sailboat) and semantically opaque (e.g., bootleg) compounds undergo morphological decomposition during the earlier stages of lexical processing, subsequent combinatorial operations should account for the difference in the contribution of the constituent morphemes to the meaning of these different word types. In this study we use magnetoencephalography (MEG) to pinpoint the neural bases of this combinatorial stage in English compound word recognition. MEG data were acquired while participants performed a word naming task in which three word types, transparent compounds (e.g., roadside), opaque compounds (e.g., butterfly), and morphologically simple words (e.g., brothel) were contrasted in a partial-repetition priming paradigm where the word of interest was primed by one of its constituent morphemes. Analysis of onset latency revealed shorter latencies to name compound words than simplex words when primed, further supporting a stage of morphological decomposition in lexical access. An analysis of the associated MEG activity uncovered a region of interest implicated in morphological composition, the Left Anterior Temporal Lobe (LATL). Only transparent compounds showed increased activity in this area from 250 to 470 ms. Previous studies using sentences and phrases have highlighted the role of LATL in performing computations for basic combinatorial operations. Results are in tune with decomposition models for morpheme accessibility early in processing and suggest that semantics play a role in combining the meanings of morphemes when their composition is transparent to the overall word meaning.

## 1. Introduction

Some words are simple and some words are not. This, at first, sounds like a very trivial tautology, but the controversy over whether multi-morphemic words are simply stored in whole word form (Butterworth, 1983; Giraudo and Grainger, 2001) or always constructed from their morphemic parts (Taft, 2004) has been entertaining, provocative, and contentious in the field of lexical processing for the last 40 years. A comprehensive model of how words are both stored and retrieved requires an understanding of how form and meaning are connected, and how this connection unfolds in time in natural speech.

The potential contrast between whole-word storage and morpheme storage was first discussed in the classic affix-stripping model (Taft and Forster, 1975), which proposed that lexical access involves access to the stem of morphologically complex words. This study demonstrated that pseudo-complex words with real stems (e.g., de-*juvenate*) took longer to reject in a lexical decision task (and were often selected incorrectly as words) than pseudo-complex words with real prefixes and non-existent stems (e.g., de-*pertoire*). This was taken as evidence that the morphemes were accessed prior to lexical access and they contribute the retrieval of the lexical item in memory. With various priming paradigms, evidence has accumulated in favor of morpheme accessibility during lexical access (Marslen-Wilson et al., 1994; Rastle and Davis, 2003; Taft, 2004). This has given rise to processing models where morphological decomposition is an automatic- and necessary stage in processing for complex words (Rastle et al., 2004). Recent studies (Fiorentino et al., 2014; Semenza and Luzzatti, 2014) have looked at the stages following decomposition to see how morpheme meaning is integrated into the meaning of the complex word. Results from electrophysiology (Fiorentino et al., 2014) revealed a greater negativity for lexicalized compounds (e.g., teacup) and novel compounds (e.g., tombnote) compared to mono-morphemic words in a time window of 275–400 ms, positing a stage where morpheme meanings are combined in English compounds. These psychological models make clear predictions as to the stages and time-course of lexical access, but currently, there is a lack of evidence for the anchoring of these stages to particular areas of the brain. This study seeks to identify an area responsible for the composition of morpheme meanings. Research from the picture naming literature (Dohmes et al., 2004) suggests that there should be greater activation at this stage in processing for semantically *transparent* complex words since they exhibit greater conceptual activation, and lemma competition in addition to the effect of morphological overlap. Therefore, this area should be sensitive only to the composition within complex words whose morpheme meaning have a semantically transparent relationship to the overall meaning as compared to complex words whose morphemes do not share a semantic relationship, *opaque*.

One way to look at the lexical processing of complex words is to see if activating morphological structure can modulate the accessibility of a complex word. Some cross-modal priming studies (Marslen-Wilson et al., 1994) have shown that priming in lexical decision between words that shared a stem only occurred when the prime and target had related meanings (e.g., *departure* primed *depart* but *department* did not) while other studies (Zwitserlood, 1994) using partial-repetition priming found that priming did not depend on a semantic relationship between the prime and target. However, studies using masked priming, a subliminal priming paradigm where a prime word is preceded by a forward mask and followed by the target word (Forster and Davis, 1984), found that when manipulating semantic transparency, facilitation effects occurred for complex words regardless of whether the prime and target share the same morphological root (Longtin et al., 2003; Rastle et al., 2004; Fiorentino and Poeppel, 2007; McCormick et al., 2008). These effects did not appear for the morphologically simple words (e.g., *brothel*). Faster lexical decision times were found for complex words that can be segmented into existing morphemes, which means that masked prime/unmasked target pairs with no semantic relationship like *corner-corn* and *bootleg-boot* speeded recognition showed of the target words with magnitudes indistinguishable from pairs with a semantic relationship like *cleaner-clean* and *teacup-tea*.

Since it is generally agreed that morphological decomposition is performed for every complex word that can be exhaustively parsed into existing morphemes, research on visual word recognition should shift its focus from decomposition to the subsequent mechanisms engaged to activate the actual meaning of a complex target word. Meunier and Longtin (2007) suggested that word activation comes into play in stages, which include at least one early stage for morphological decomposition and a later stage for semantic integration of the morphological pieces. Fiorentino et al. (2014) presented evidence for a morpheme-based route for word activation that includes decomposition into morphological constituents and combinatorial processes operating on these representations. Since previous studies have shown that early decomposition triggered by morphological structure happens automatically for transparent and opaque words, the difference between these two word types may manifest itself during a later stage of combinatorial operations.

Another way to look at lexical processing of complex words is to look at how form is mapped onto meaning. This is critical in processing morphologically complex words in order to disentangle how the brain perceives transparent ones from how it perceives opaque ones. This can be investigated by looking at how morpheme meanings are composed in the brain. There are models for a general binding mechanism in sentence building (Friederici et al., 2000) and in basic composition of noun phrases (Bemis and Pylkkänen, 2011) that implicate the left Anterior Temporal Lobe (LATL) in the composition of words into phrases. In a minimum composition paradigm, Bemis and Pylkkänen (2011) found that two composable items in an adjective-noun phrase (e.g., *red boat*) evoked more activation in the left anterior temporal lobe, LATL, at roughly 225 ms, than two non-composable items (e.g., *xkq boat*, a random letter string and word). This was taken as evidence that the most basic of combinatorial processing is supported by the LATL. Within complex words, there is a special subclass of words that have a parallel structure to noun phrases known as compound words. Compound words have the unique property of being composed of only free morphemes (stand-alone words). Compound words also vary along the dimension of *semantic transparency*, the degree to which the combination of morpheme meanings corresponds to the overall word meaning. This means we can vary the contribution of the morphemes to the composition of the meaning. These properties make compound words a great candidate for investigating morphological composition within complex words since they can provide an analogous structure to work done at the phrase level. These parallels give rise to the LATL as a candidate region for composition within a word and this provides an interesting basis for studying effects of intra-lexical semantic composition as an analog to composition at the phrase level.

Thus, semantically transparent compound words (e.g., mailbox) should elicit greater activity in this region than simple words since their meanings are derived from the composition of their morphemic parts, whereas semantically opaque compounds (e.g., bootleg) should not elicit greater activity since there is no relationship between their parts and meanings. In sum, a model of complex word recognition would require at least these two stages of processing: parsing into basic units (decomposition), and the composition of these word forms into a complex meaning. To unpack these stages, we propose using two types of priming paradigms: partial-repetition priming (e.g., ROAD-roadside), similar to the paradigms used in masked priming studies, which will be used to investigate the decomposition effects in compounds, and a full-repetition priming (e.g., ROADSIDE-roadside), which will be used to investigate the composition effects of their morphemes. The primes of the repetition priming condition were used to evaluate the composition effect in the absence of a behavioral response. In this respect, the method of analysis analogous to that adopted by Zweig and Pylkkänen (2009), in which the authors directly compare complex (derived) words, thus aiming to find decomposition effects that are not dependent on priming. This study uses a word naming production task to investigate these stages involved in lexical processing since it provides comparable effects to lexical decision tasks (Neely, 1991) and does not require filler trials. This task was done while brain activity was recorded using MEG to investigate whether there is an area within the left temporal lobe that is responsible for morphological composition. This study contributes to the work of characterizing the neural bases of lexical processing of complex words by providing evidence for composition within compound words, while linking it to their neural correlates. Given the prior literature, we expect to find evidence of decomposition for compound words but not for simplex words. This would be a finding that fits in with the visual word recognition literature, specifically the masked priming literature, where there are facilitatory effects when priming morphologically complex words but not morphologically simple words. However, we do not expect to find this overall benefit of morphological complexity in composition. Since composition of meaning is semantically governed, we expect to find composition effects on brain activity only for transparent compounds.

## 2. Materials and methods

### 2.1 Participants

Eighteen right-handed native speakers of English ranging from 18 to 30, with normal or corrected vision, all gave informed consent and participated in this experiment. The study was approved by the University Committee on Activities Involving Human Subjects (UCAIHS) of New York University. The MEG data from three participants were excluded due to the large number of trial rejections caused by a noise interference (>25%). Details for rejection are described in the procedure.

### 2.2. Material

All stimuli consisted of English bi-morphemic compounds (e.g., teacup) and morphologically simple (e.g., spinach) nouns, matched for length and surface frequency. We manipulated semantic transparency, including fully semantically transparent (e.g., teacup) words, in which both constituent morphemes have a semantic relationship to the meaning of the whole compound, and fully semantically opaque words (e.g., hogwash), in which neither of the constituent morphemes have a semantic relationship to the compound meaning.

311 English compounds were compiled from previous studies (Juhasz et al., 2003; Fiorentino and Poeppel, 2007; Fiorentino and Fund-Reznicek, 2009; Drieghe et al., 2010) and categorized in terms of semantic transparency by means of a semantic relatedness task conducted using the Amazon Mechanical Turk tool. In this task, 20 participants were asked to judge, on a 1–7 scale, how much each constituent of the compounds related to the whole word. On the scale, 1 corresponded to unrelated and 7 corresponded to very related. Each participant was randomly presented with one of the constituents of each compound. Compounds were classified as semantically opaque (henceforth *opaque*) if the sum of the scores of their constituents was within the interval 2–6, and as semantically transparent (henceforth *transparent*) if the sum were within the interval 10–14. For example, the opaque compound *deadline* received a summed rating of 3.76 with *dead* contributing a transparency rating of 1.44 and *line* contributing a rating of 2.32. Similarly, the compound *dollhouse* received a summed rating of 11.79 with *doll* contributing a transparency rating of 6.47 and *house* contributing a rating of 5.32. Sixty compounds were selected for each word type. This method of semantic transparency norming was consistent with the methods used in the mentioned prior studies. The morphologically simple words (henceforth *simplex*: e.g., spinach) were pooled from Rastle et al. (2004) and the English Lexicon Project selecting the words coded for having only one morpheme (Balota et al., 2007). The simplex words (e.g., *brothel*) were selected to have a non-morphological form relationship to their primes (e.g., *broth*). Also, these words were constrained and selected such that the simple word could not be broken into smaller parts without creating illegal morphemes.

### 2.3. Design

The three different word types were contrasted in two priming conditions: full repetition and partial (constituent) repetition (See Table 1). For the repetition priming condition, the same compound was used as prime and target (e.g., TEACUP-teacup). For the partial-repetition priming, we used the first constituent of the compound as the prime (e.g., TEA-teacup). For the simplex condition, the non-morphological related form was used as the constituent in the partial-repetition priming condition (e.g., SPIN-spinach). These two priming conditions were paired to control conditions in which the prime had no semantic relationship to the target (e.g., DOORBELL-teacup; DOOR-teacup).

**Table 1:** **Design matrix**.

	**Transparent**		**Opaque**		**Simplex**	
	**Prime**	**Target**	**Prime**	**Target**	**Prime**	**Target**
Control	Doorbell	Teacup	Heirloom	Hogwash	Brothel	Spinach
Repetition	Teacup	Teacup	Hogwash	Hogwash	Spinach	Spinach
Control	Door	Teacup	Heir	Hogwash	Broth	Spinach
Partial-repetition	Tea	Teacup	Hog	Hogwash	Spin	Spinach

### 2.4. Procedure

**Figure 1:**
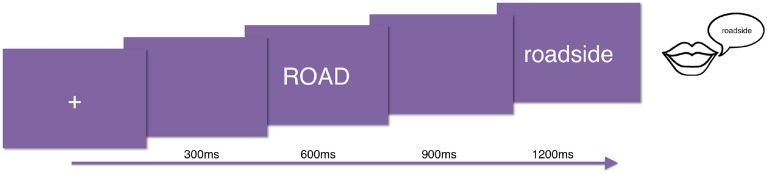
**Experiment trial structure**.

All participants read all the items in all conditions (720 total), which were divided in three lists of 240 words and randomized within each list. The order of presentation of the lists was counterbalanced between subjects. The experimental task was word naming: subjects were presented with word pairs, and they were asked to read out loud the second word of each pair. Stimuli were presented in 30-point white Courier font on a gray background using PsychToolbox (Brainard, 1997). Each trial began with the presentation of a fixation cross, followed by the prime, then the target. Each of these visual presentations was presented for 300 ms followed by a 300 ms blank (see Figure 1). We recorded the onset latency to speech and the utterance from each subject for behavioral analysis.

Before the experiment, the head shape of each participant was digitized using the Polhemus Fastscan system, along with five head position indicator points, which are used to co-register the head position with respect to the MEG sensors during acquisition. Electromagnets attached to these points are localized after the participants are lying within the MEG sensor array, allowing for co-registration of head and sensor coordinate systems. The head shape is used during the analysis to co-register the head to participants MRIs. For half of the participants, MRIs were not provided; therefore, we scaled the common reference brain that is provided in FreeSurfer to fit the size of these participants' heads.

During the experiment, participants remained lying in a magnetically shielded room as their brain response was monitored by the MEG gradiometers. The experimental items were projected onto a screen so the participant could read and perform the task. The MEG data were collected using an axial whole-head gradiometer system with 157 channels and three reference channels (Kanazawa Institute of Technology, Nonoichi, Japan). The recording was conducted in direct current mode, that is, without a high-pass filter, and with a 300 Hz low-pass filter and a 60 Hz notch filter.

### 2.5. Analysis

We examined onset latency, the reaction time to naming the word, to evaluate the effects of morphological decomposition based on Fiorentino and Poeppel (2007). Since reaction time is sensitive to lexical properties of words (Fiorentino and Poeppel, 2007), compound words should be processed faster when primed than simplex words due to residual activation of previously activated morphemes. A non-decompositional account predicts no differences due to word structure, if the words are correctly matched for relevant whole word properties. Thus, onset latency can be used to disentangle whether or not there is a decomposition effect. The behavioral data were analyzed using traditional analysis of variance for the Word Type by Partial-Repetition priming interaction model. Partial-repetition priming in lexical decision tasks has been used to demonstrate the accessibility of morphemes within complex words (Rastle et al., 2004). Similar behavioral effects have also been found using word naming (see Neely, 1991 for a comparative review of lexical decision and word naming). Therefore, the evidence of decomposition effects can be observed in the reaction time to speak, *onset latency*. Prior research led to the prediction that there should be a facilitative effect of shorter onset latency due to priming for the compounds as compared to their simplex word counterparts since the segmentation into morphemes lead to faster access to the complex word.

After brain data acquisition, we applied a Continuously Adjusted Least-Squares Method (Adachi et al., 2001), a noise reduction procedure in the MEG160 software (Yokogawa Electric Corporation and Eagle Technology Corporation, Tokyo, Japan) that subtracts noise from the MEG gradiometers based on noise measurements at the reference channels positioned away from the head. The data were bandpass filtered between 1–40 Hz using an IIR filter. The recording of the whole experiment was segmented into epochs of interest, from −200 ms before to 600 ms after the visual display of the prime word. We rejected trials in which the maximal peak-to-peak amplitude exceeded the limit of 4000fT and we equalized the trials to have an equal number of trials per condition and per word type for proper comparison. The average percentage of all trials rejected across subjects was 1.9%, and per word type: 1.3% for opaque, 2.2% for simplex, 1.8% for transparent. Sensor channels were marked as bad and discarded for each subject if the channel's peak-to-peak rejection exceeded 10%.

A noise-covariance matrix was computed for each participant using an automated model selection procedure (Engemann and Gramfort, 2015) on a random selection of baseline epochs (120 epochs) from −200 ms to the onset of the presentation of the fixation cross. For participants with MRIs, cortical reconstructions were generated using FreeSurfer resulting in a source space of 5124 vertices (CorTechs Labs Inc., La Jolla, CA and MGH/HMS/MIT Athinoula A. Martinos Center for Biomedical Imaging, Charleston, MA). A boundary-element model (BEM) method was used to model activity at each vertex to calculate a forward solution. An inverse solution was generated using this forward model and noise-covariance matrix, and was computed with a fixed-orientation constraint requiring dipole sources to be normal to the cortical surface. The sensor data for each subject was then projected into their individual source space using a cortically-constrained minimum norm estimate (all analyses were conducted using MNE-Python: Gramfort et al., 2013, 2014) resulting in noise-normalized dynamic statistical parameter maps (dSPMs: Dale et al., 2000).

For this analysis, our design (Table 2) reduces to the simple comparison between compounds (e.g., TEACUP) and simplex words (e.g., SPINACH) of the same size that served as primes in the repetition condition (e.g., TEACUP-teacup) described above in the Design section. Since, for this analysis, we use neurophysiological data related to the silent reading of the words that served as primes, there is no behavioral data for these words. By these means we also avoid artifacts associated with voluntary movements that can compromise the analysis of the effects of interest to the study (Hansen et al., 2010).

**Table 2:** **Primes analysis**.

**Word types**	**Examples**
Opaque	Hogwash
Transparent	Teacup
Simplex (control)	Brothel

We examined the neural activity localized in the entire left temporal lobe. This region was selected based on composition effects found with sentences (Friederici et al., 2000) or adjective-noun phrases (Bemis and Pylkkänen, 2011). In order to verify if there was increased activity for compounds in this area, a *t*-test was performed on the residual activation of a compound word type (opaque, transparent) after removing the activation from the simplex control word from 100 to 600 ms after the stimulus onset. The *p*-value map of the brain was generated for the time series and spatiotemporal clusters were identified for contiguous space-time clusters that had a *p*-value of less than 0.05 and a duration of at least 10 ms. The *t*-values were summed for those points within the cluster that met these criteria. Then, a non-parametric permutation test was performed first by shuffling the word type labels, then calculating clusters formed by the new labels. A distribution generated from 10,000 permutations was computed from calculating significant levels of the observed cluster. The corrected *p*-value was determined from the percentage of clusters that were larger than the original computed cluster (Maris and Oostenveld, 2007). These tests were computed using the statistical analysis package for MEG data, Eelbrain, (https://pythonhosted.org/eelbrain/).

## 3. Results

### 3.1. Morphological decomposition

**Figure 2:**
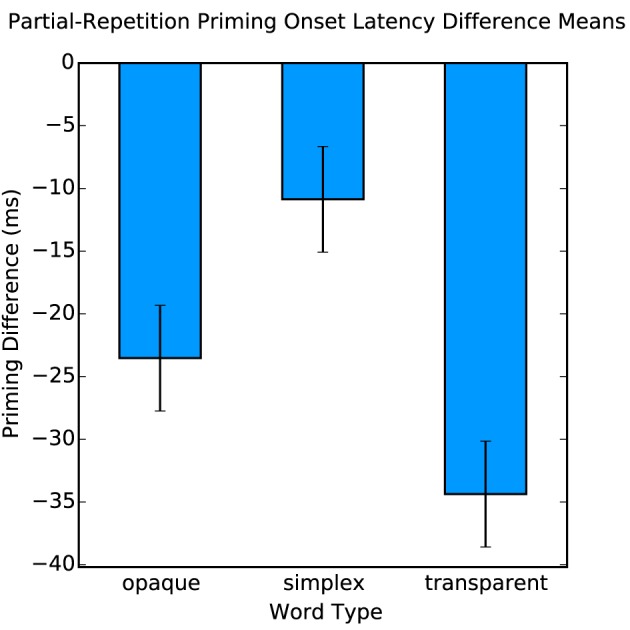
**Partial-repetition priming onset latency difference means**.

Behaviorally, we found a significant effect of partial-repetition priming [*F*_(1, 17)_ = 25.91, *p* < 0.001], but most critically an interaction of word type by priming [*F*_(2, 17)_ = 9.24, *p* < 0.001] (Figure 2). This effect shows that there is a greater facilitation in word naming for compound words than for morphologically simple words when primed. In the planned comparisons, reliable differences were found between opaque compounds and simplex words [*F*_(1, 17)_ = 5.93, *p* < 0.03], and transparent compounds and simplex words [*F*_(1, 17)_ = 14.46, *p* < 0.005] but not between transparent and opaque compounds [*F*_(1, 17)_ = 2.84, *p* > 0.1]. These results show that even in word production, there is sensitivity to morphological structure above and beyond orthographic and phonological overlap, but this stage of processing is not sensitive to the meaning of the morphemes in relationship to the compound word, which is consistent with the prior literature on morphological decomposition (Rastle et al., 2004; McCormick et al., 2008).

### 3.2. Morphological composition

**Figure 3:**
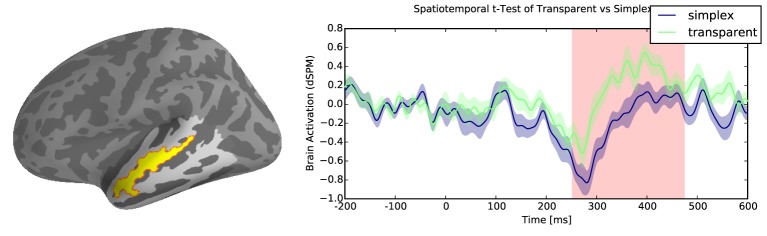
**Transparent vs. simplex difference in Left Anterior Temporal Lobe (LATL)**.

**Figure 4:**
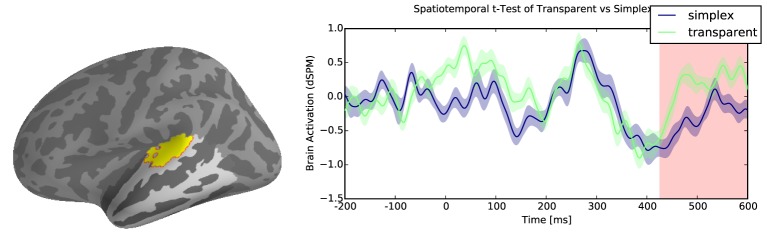
**Transparent vs. simplex difference in Posterior Superior Temporal Gyrus (pSTG)**.

Results reveal reliable effects of greater activation for transparent compounds when compared with their simplex controls within the temporal lobe. There were two significant clusters associated with this difference: the first cluster was localized to the anterior middle temporal gyrus from 250 to 470ms (∑ *t* = 4552.3, *p* < 0.05, Figure 3), and a second cluster of activity was localized to the posterior superior temporal gyrus from 430 to 600 ms (∑ *t* = 5654, *p* < 0.05, Figure 4). However, there were no reliable clusters found for the difference of opaque compounds and simplex words within the temporal lobe.

## 4. Discussion

Analyses of the different word types in isolation revealed very consistent evidence that there is a difference in how simplex and complex words are processed in the brain. The behavioral results confirmed that there is a stage in lexical access that is sensitive to the morphological forms within complex words and demonstrated that these effects could also be observed in other testing modalities, namely, word naming. The onset latency interaction effect where compound words were faster to produce than morphologically simple words when primed by their constituent morpheme is largely consistent with the results within the masked priming literature on word recognition, and gives further evidence that there is a decomposition stage in lexical access where complex words are parsed into their morphemes (Rastle et al., 2004; Taft, 2004; Morris et al., 2007; McCormick et al., 2008; Fiorentino and Fund-Reznicek, 2009). The parsing operation occurs independent of the semantic relationship between constituent morphemes and their complex word. Since early activation of constituents via morphological decomposition happens irrespective of semantic transparency, what differentiates transparent and opaque compound must happen, thus, during a later stage of morphemic composition. The increased activity found for transparent compounds in anterior temporal lobe from 250 to 470ms provides evidence for a stage in lexical access where meanings of the morpheme play a part in accessing the overall meaning of the word. Bemis and Pylkkänen (2011) show combinatorial effects in the LATL for adjectival words at around 225 ms after the critical word is presented. The difference in timing could be explained by the different time points at which we time lock the onset of the stimulus. In Bemis and Pylkkänen (2011), the onset coincides with the onset of the noun *boat* in the phrase *red boat*, whereas in our study the critical stimulus is the entire compound *sailboat*.

The increased activation in the posterior temporal lobe for transparent compounds from 430 to 600 ms that follows the activity in the LATL is consistent with the fact that this region is involved in lexical retrieval (Hickok and Poeppel, 2007; Lau et al., 2008). Lau et al. (2008) proposed that the posterior region of the temporal lobe is the best candidate for the lexical storage of words. Since the LATL is responsible for composing the meaning of the constituent morphemes, the posterior temporal lobe would be responsible for retrieving information from its stored lexico-semantic representation. This region is also engaged in sound-to-meaning transformation (Binder et al., 2000), which would include the retrieval of phonological information. This study is in tune with decomposition models from visual word recognition literature and provides the neural basis for a stage in lexical access involved in the composition of meaning within compound words, thus helping to disentangle cognitive processes that are indistinct when reaction time is the only measure. Bridging results from psycholinguistic research with MEG recordings of brain activity, the emerging results suggest that the recognition of compounds involves distinct stages: a decomposition stage that is independent of semantics, and a composition stage that is governed by semantics. We showed that the course of activation varies in terms of word complexity and semantic transparency.

## Author contributions

Authors TB and DC share first-authorship as they have both equally contributed to the paper.

### Conflict of interest statement

The authors declare that the research was conducted in the absence of any commercial or financial relationships that could be construed as a potential conflict of interest.
